# Publication Trends on Mitophagy in the World and China: A 16-Year Bibliometric Analysis

**DOI:** 10.3389/fcell.2021.793772

**Published:** 2021-11-29

**Authors:** Jingli Chen, Xin Li, Yifan Jia, Zhongyuan Xia, Jishi Ye

**Affiliations:** ^1^ Department of Pain, Renmin Hospital of Wuhan University, Wuhan, China; ^2^ Department of Anesthesiology, The Central Hospital of Wuhan, Tongji Medical College, Huazhong University of Science and Technology, Wuhan, China; ^3^ Department of Anesthesiology, Hubei Cancer Hospital, Tongji Medical College, Huazhong University of Science and Technology, Wuhan, China

**Keywords:** mitophagy, bibliometric analysis, VOSviewer, web of science, gap

## Abstract

In the past 16 years, research on mitophagy has increasingly expanded to a wider range of subjects. Therefore, comprehensively analyzing the relevant progress and development trends on mitophagy research requires specific methods. To assess the hotspots, directions, and quality of results in this field worldwide, we used multiple tools to examine research progress and growing trends in research on the matter during the last 16 years (from 2005 to 2020). We also compared the quantity and quality of the literature records on mitophagy published by research institutions in China and other developed countries, reviewed China’s contribution, and examined the gap between China and these developed countries. According to the results of our bibliometric analysis, the United States and its research institutes published the most papers. We identified cell biology as the most commonly researched subject on mitophagy and AUTOPHAGY as the most popular journal for research on mitophagy. We also listed the most cited documents from around the world and China. With gradually increased funding, China is progressively becoming prominent in the field of mitophagy; nevertheless, the gap between her and major countries in the world must be closed.

## Introduction

Mitochondria, complex organelles in the cell, keep complex organisms alive and are important biochemical metabolism centers of cells (Zilocchi et al., 2019). A mitochondrion produces adenosine triphosphate through oxidative phosphorylation and provides a key intermediate for the biosynthesis of amino acids, hormones, and fatty acids. Cells develop complex and interconnected regulatory pathways to maintain mitochondrial homeostasis by balancing mitochondrial biogenesis and clearing out damaged mitochondria. Therefore, signaling pathways that regulate mitochondrial homeostasis are crucial to cell survival, and their dysfunction is closely associated with aging and the occurrence and development of major diseases, such as cancer, cardiovascular, and neurodegenerative diseases (Pamenter et al., 2018; Iwata, 2019).

Mitophagy is a form of autophagy that mediates the removal of defective or redundant mitochondria (Doxaki and Palikaras 2020; Wang et al., 2021). And it is the only known way to selectively remove entire mitochondria (D'Acunzo et al., 2019). Mitophagy can also inhibit the production of excessive reactive oxygen species and retain valuable nutrients (such as oxygen) so that they are not consumed inefficiently, thus promoting cell survival under various pathological conditions (Cai et al., 2012; Salimi et al., 2019; Ye et al., 2019).

In the past decade, more and more investigations have been conducted on mitophagy. Therefore, comprehensively analyzing the relevant progress and development trend in the field of mitochondrial autophagy requires specific methods. Bibliometrics, an analysis technique based on the number of citations and citation models, can be used to evaluate the impact of various research findings, such as a journal article or the number of citations and research directions of a series of research results (Bernard et al., 2019; Chang et al., 2019; Garcovich and Adobes Martin, 2019). Before being employed in this manner, bibliometric analysis was widely used to assess progress and trends in different research fields, including anesthesiology (Ye et al., 2018), critical care medicine (Nakao et al., 2019), and urology (Zhao et al., 2019), and played an essential role in understanding current research hotspots and future trends.

Up to now, there have been no bibliometric reports on research trends and scientific outcomes in the field of mitophagy. VOSviewer is a software tool developed based on the Java programming language for building and visualizing bibliometric networks. These networks may include journal researchers or individual publications constructed using citation lists coupled to common citations or co-author relationships. VOSviewer also provides text mining capabilities for building and visualizing co-existing networks of important terms extracted from the scientific literature. This software can be used to build networks of scientific publications, scientific journals, and researchers who study and organize national keywords or terms. Moreover, VOSviewer provides three types of visual maps: network visualization, overlay visualization, and density visualization, which can enable the further understanding of the relationships between institutions, authors, and keywords (Lei et al., 2019).

To assess the hotspots, directions, and quality of mitophagy-related results worldwide, we used bibliometrics to analyze research progress and development trends in this field during the last 16 years (from 2005 to 2020). We also compared the quantity and quality of the literature records in the field published by research institutions in China and other developed countries, reviewed China’s contribution, and examined the gap between China and these developed countries.

## Methods

### Database

This research was carried out in the same way as previous similar literature (Wang et al., 2019). All data were collected on January 28, 2021: data were downloaded from public databases. There were no ethical issues, with ethical approval not applicable for this study. Articles on mitophagy published from 2005 to 2020 were retrieved from PubMed and web of science (WOS) online databases. Impact factors (IF) were obtained from the citation report database of journals in 2019.

### Retrieval Strategy

The search terms in WOS were as follows: TOPIC: (mitophagy), Refined by: DOCUMENT TYPES: (ARTICLE OR EDITORIAL MATERIAL OR REVIEW OR PROCEEDINGS PAPER OR MEETING ABSTRACT OR LETTER) AND LANGUAGES: (ENGLISH), Databases = WOS, BIOSIS, INSPEC, KJD, MEDLINE, RSCI, SCIELO Timespan = 2005–2020.

The search terms in PubMed were as follows: (“mitophagy” [MeSH Terms] OR “mitophagy” [All Fields]) AND (bibliography [Filter] OR clinical study [Filter] OR journal article [Filter] OR letter [Filter] OR review [Filter]).

### Bibliometric Analysis

Using the intrinsic function of WOS, we analyzed the trends in research publication and the quality of publications from 2005 to 2020. Factors scrutinized included country/region, institution distribution, and top 10 cited references with high citation frequency.

We also used VOSviewer (Van Eck& Waltman, Leiden University, Netherlands) and Microsoft Excel to mine, map, and cluster data from the retrieved articles. The programs portrayed keywords and countries using various colors and sizes of circles based on the frequency of occurrence of a keyword or country in both the titles and abstracts.

## Results

### Trends in Publications on Mitophagy in the World and China

**FIGURE 1:**
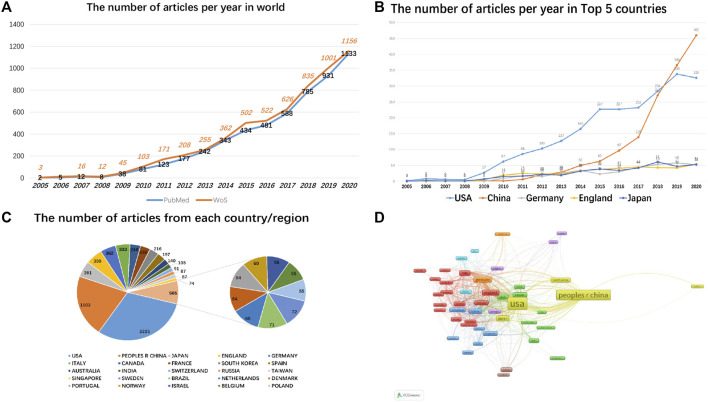
Mitophagy-related article overview. **(A)** The number of mitophagy publications across the world from WOS and PubMed database. **(B)** The time curve of mitophagy-related articles from the top 5 countries. **(C)** The number of articles from each country/region. **(D)** A network map showing the collaborative relationships between various countries in the field of mitophagy research.

From 2005 to 2020, a total of 5,829 and 5,338 papers on mitophagy were recorded in the WOS and PubMed databases, respectively, based on the above retrieval strategy. Because the included journals from the two databases are different in content, the results are unlikely to be similar. The number of related papers published each year shows a significant upsurge year after year, highlighting a gradually increasing interest in mitophagy over the past 16 years. Our inquiry revealed that researchers from 137 countries were involved in scientific studies on mitochondrial autophagy. United States-based investigators published the most papers (2,225, 38.171%) in the WOS database, followed by mainland China (1,503, 25.785%), Japan (390, 6.691%), England (391, 6.708%), and Germany (362, 6.210%). Among the top five countries with the largest amount of publications, the United States and mainland China experienced the most significant rise (Figures 1A–C).

Using the VOSViewer software, we analyzed the cooperative relationships between all countries in the field of mitophagy. Per our findings, only 51 countries had published more than five papers at the time of our analysis, while there were 8 close relationships. Among them, the United States, mainland China, Japan, and South Korea cooperated closely. England, Portugal, Brazil, and Iran worked closely together too. Germany had frequent associations with Luxembourg, Sweden, Netherlands, and Ireland. Therefore, *trans*-national and *trans*-regional communication in the field of mitophagy is quite common. Combined with growth curves and collaboration networks, the United States remains at the center of the field, while China is playing an increasingly key role.

### Citation of Related Papers and H-Index Analysis

From the perspective of institutions with relevant publications, the University of Pittsburgh, the University of California - San Diego, Chinese Academy of Sciences, University College London, and Fudan University have published the most papers on mitophagy in the world: the University of Pittsburgh leads the way in this category and had the highest H-index and total citations, reflecting its prominent position in the field. Its paper “Loss of PINK1 Function Promotes Mitophagy through Effects on Oxidative Stress and Mitochondrial Fission”, written by Dagda, Ruben K, and coauthors (Dagda et al., 2009) and published in 2009, was cited 615 times.

While the number of papers from Chinese organizations has increased rapidly, a gap remains between them and the world’s top-tier countries and institutions. Among mainland Chinese establishments, the three institutions with the most published papers on mitophagy are the Chinese Academy of Sciences, Fudan University, and Zhejiang University. The Chinese Academy of Sciences has published up to 107 papers: its “Mitochondrial outermembrane protein FUNDC1 mediates hypoxia-induced mitophagy in mammalian cells”, written by Liu, Lei, and coauthors (Liu et al., 2012) and published in 2012, has received 635 citations.

**FIGURE 2:**
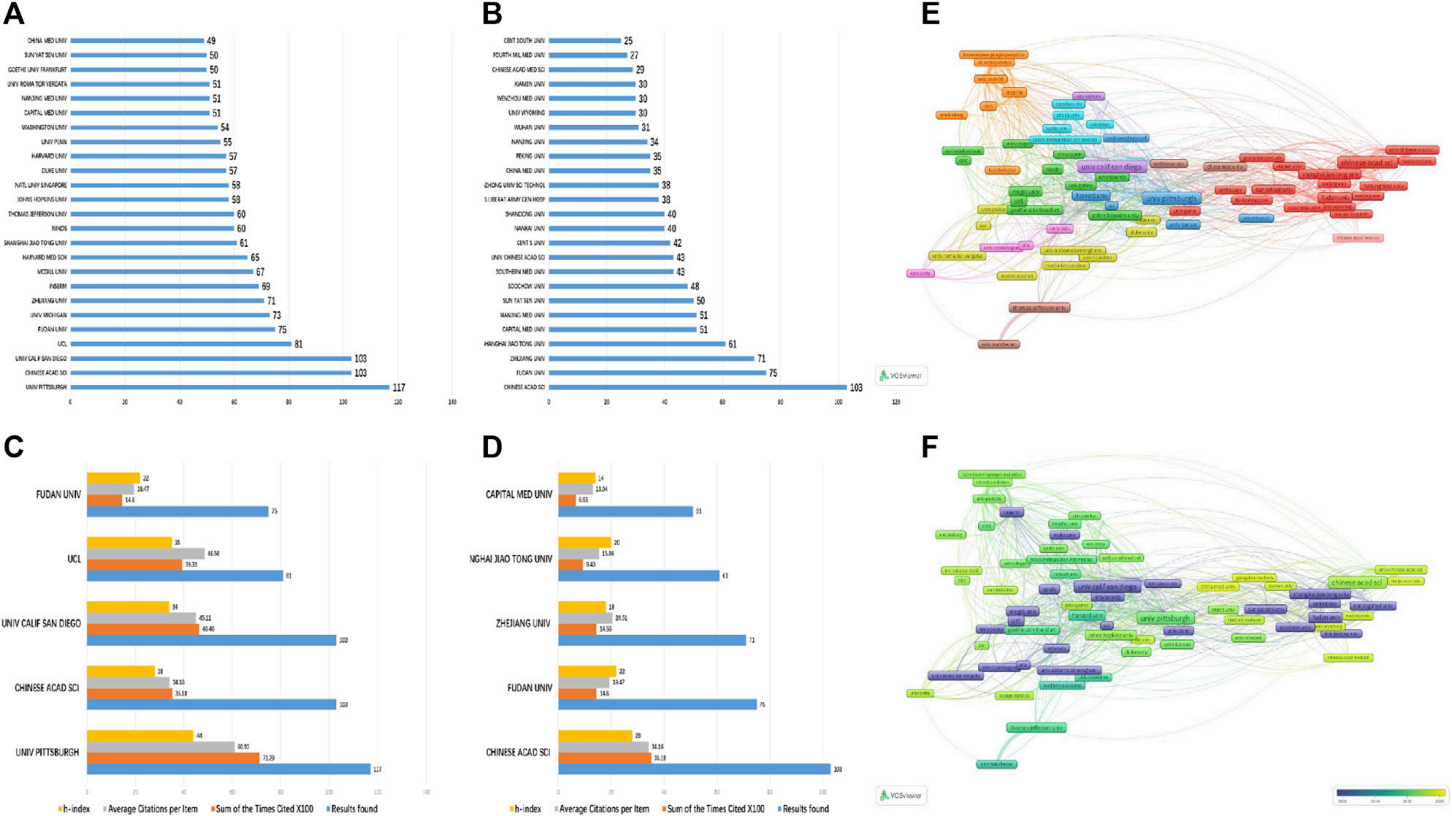
Citations of related papers and H-index analysis. **(A)** The number of mitophagy publications from institutions across the world. **(B)** The number of mitophagy publications from Chinese institutions. **(C)** The total citations, average citations per paper, and H-indexes for mitophagy articles from institutions across the world. **(D)** The total citations, average citations per paper, and H-indexes for neuropathic pain articles from Chinese institutions. **(E)** A network map showing the collaborative relationships between various institutions in the field of mitophagy research. **(F)** A density map showing the collaborative relationships between various institutions in the field of mitophagy research.

Using the VOSViewer software, we identified about 609 institutes with more than 5 published papers on mitophagy and about 137 institutes with more than 20 published papers. As shown in Figures 2E,F, our co-occurrence chart on research institutions revealed that the University of Pittsburgh has a close working relationship with some research establishments in China, including the Chinese Academy of Sciences, Central South University, and Zhejiang University, while Harvard University frequently collaborates with other American universities, like the University of California - San Diego. Per the coverage visualization chart, the most prominent research institutions on mitophagy have gradually shifted from European and American universities to research establishments in mainland China (Figures 2E,F).

### Research Direction in the Field of Mitophagy and Distribution of Journals

**FIGURE 3:**
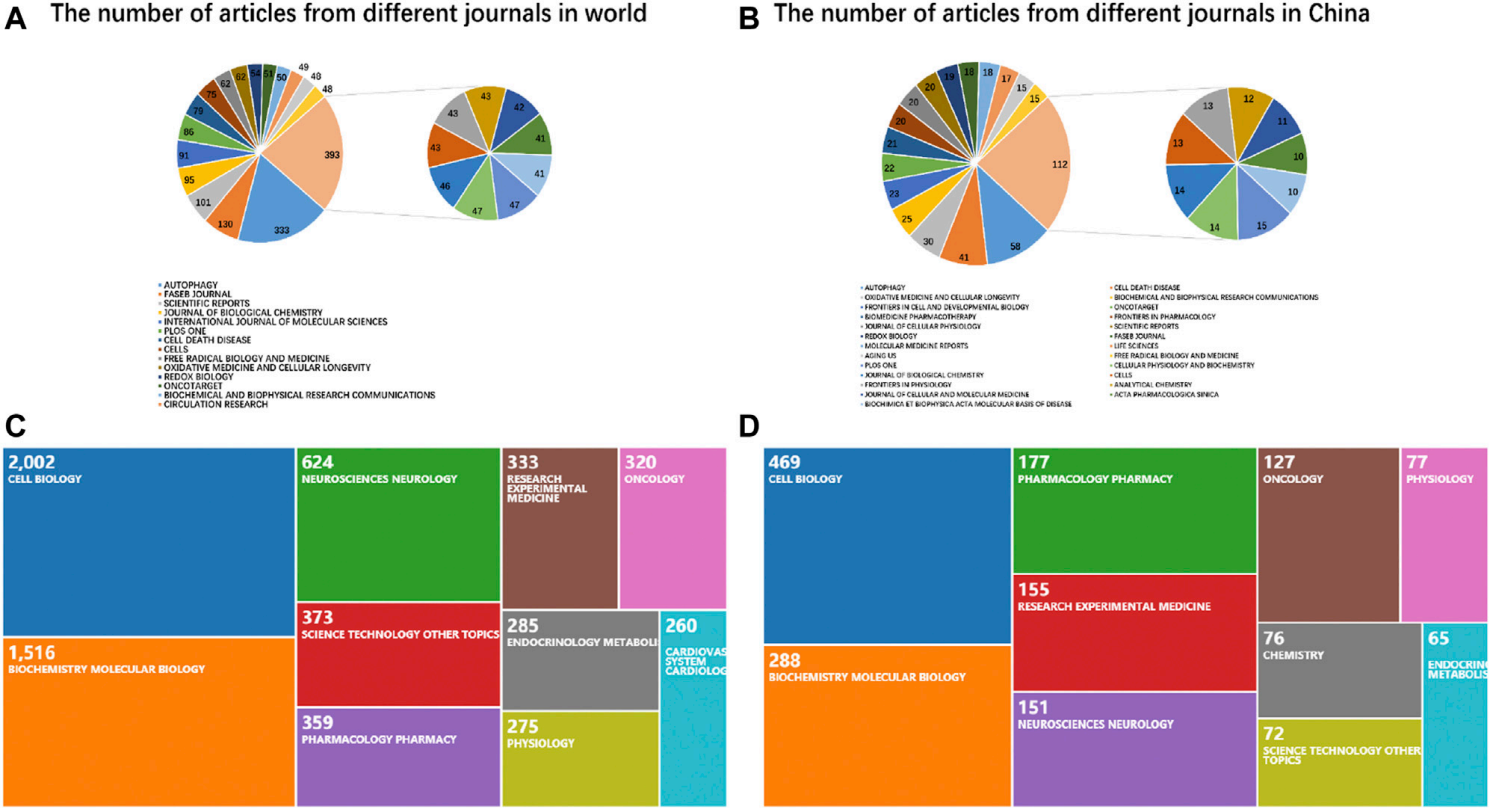
Research direction in the field of mitophagy and the distribution of journals. **(A)** The number of articles from different journals across the world. **(B)** The number of articles from different journals in China. **(C)** Research direction in the field of mitophagy worldwide. **(D)** Research direction in the field of mitophagy in China.

Globally, AUTOPHAGY has published the most papers on mitophagy (333, 5.713%) as of the time of our research, followed by FASEB JOURNAL (130, 2.23%) and SCIENTIFIC REPORTS (101, 1.733%). According to the Q1 classification of Journal Citation Reports (2019 version), most of the top ten publishing journals were not among the very top journals in the world. China has a similar situation (Figures 3A,B).

In terms of research direction, investigations on mitophagy around the world focused on Cell Biology, Biochemistry, Molecular Biology, Neurosciences, Neurology, Science Technology, Other Topics, and Oncology. Comparable to the research direction around the globe, research direction on mitophagy in mainland China also focuses on Cell Biology, Biochemistry, Molecular Biology, Oncology, Neurosciences, Neurology, and Pharmacology Pharmacy (Figures 3C,D).

### The Top 10 Most Cited Articles on Mitophagy in the World and China

Using the built-in tools in the WOS database, we screened out the most cited papers with mitophagy-related subjects in the world and China, as shown in Table 1, 2. The two most cited articles abroad were reviews published in top journals. The paper “Pink1/Parkin-mediated mitophagy is dependent on VDAC1 and P62/SQSTM1”, written by Geisler et al. (2010), was the most cited research paper in the past 15 years. Parkinson’s disease, the most common neurodegenerative movement disorder, was the subject of discourse in this paper. Current research has identified PINK1 and Parkin mutations as the most common causes of Parkinson’s disease. However, their molecular roles in the pathogenesis of the disease remain unclear. Per the investigation, the PINK1/Parkin pathway is a critical mechanistic step linking mitochondrial damage to ubiquitination and autophagy in non-neurons and neuronal cells. PINK1 kinase activity and its mitochondrial localization sequence are the prerequisites for inducing the translocation of E3 ligase Parkin to depolarized mitochondria. Notably, Geisler et al. identified voltage-dependent anion channel 1 (VDAC1) as a target for Parkin-mediated Lys 27 polyubiquitination and mitophagy. Overall, the paper established a functional association between Pink1-Parkin and mitochondrial selective autophagy, which is involved in the pathogenesis of Parkinson’s disease.

**TABLE 1:** The top 10 most cited articles on mitophagy research in the world.

Title	Authors	Total citations	Average per year	Source title	Publication date
A role for mitochondria in NLRP3 inflammasome activation	Zhou, Rongbin, et al.	2,477	225.18	NATURE	JAN 13 2011
Mechanisms of mitophagy	Youle, Richard J., et al.	1,680	152.73	NATURE REVIEWS MOLECULAR CELL BIOLOGY	JAN 2011
PINK1/Parkin-mediated mitophagy is dependent on VDAC1 and p62/SQSTM1	Geisler, Sven, et al.	1,589	132.42	NATURE CELL BIOLOGY	FEB 2010
PINK1 Is Selectively Stabilized on Impaired Mitochondria to Activate Parkin	Narendra, Derek P., et al.	1,554	129.5	PLOS BIOLOGY	JAN 2010
Phosphorylation of ULK1 (hATG1) by AMP-Activated Protein kinase Connects Energy Sensing to Mitophagy	Egan, Daniel F., et al.	1,434	130.36	SCIENCE	JAN 28 2011
Selective degradation of mitochondria by mitophagy	Kim, Insil, et al.	1,032	68.8	ARCHIVES OF BIOCHEMISTRY AND BIOPHYSICS	JUN 15 2007
PINK1 stabilized by mitochondrial depolarization recruits Parkin to damaged mitochondria and activates latent Parkin for mitophagy	Matsuda, Noriyuki, et al.	1,024	85.33	JOURNAL OF CELL BIOLOGY	APR 19 2010
PINK1-dependent recruitment of Parkin to mitochondria in mitophagy	Vives-Bauza, Cristofol, et al.	973	81.08	PROCEEDINGS OF THE NATIONAL ACADEMY OF SCIENCES OF THE UNITED STATES	JAN 5 2010
The ubiquitin kinase PINK1 recruits autophagy receptors to induce mitophagy	Lazarou, Michael, et al.	936	133.71	NATURE	AUG 20 2015
Mitochondrial dynamics-fusion, fission, movement, and mitophagy-in neurodegenerative diseases	Chen, Hsiuchen, et al.	868	66.77	HUMAN MOLECULAR GENETICS	OCT 15 2009

**TABLE 2:** The top 10 most cited articles on mitophagy research in China.

Title	Authors	Total citations	Average per year	Source title	Publication date
Mitochondrial outer-membrane protein FUNDC1 mediates hypoxia-induced mitophagy in mammalian cells	Liu, Lei, et al.	636	63.6	NATURE CELL BIOLOGY	FEB 2012
Hepatic Autophagy Is Suppressed in the Presence of Insulin Resistance and Hyperinsulinemia INHIBITION OF FoxO1-DEPENDENT EXPRESSION OF KEY AUTOPHAGY GENES BY INSULIN	Liu, Hui-Yu, et al.	247	19	JOURNAL OF BIOLOGICAL CHEMISTRY	NOV 6 2009
Cerebral ischemia-reperfusion-induced autophagy protects against neuronal injury by mitochondrial clearance	Zhang, Xiangnan, et al.	245	27.22	AUTOPHAGY	SEP 1 2013
ROS and Autophagy: Interactions and Molecular Regulatory Mechanisms	Li, Lulu, et al.	224	32	CELLULAR AND MOLECULAR NEUROBIOLOGY	JUL 2015
ULK1 translocates to mitochondria and phosphorylates FUNDC1 to regulate mitophagy	Wu, Wenxian, et al.	217	27.13	EMBO REPORTS	MAY 2014
Mitochondrial Regulation in Pluripotent Stem Cells	Xu, Xiuling, et al.	215	23.89	CELL METABOLISM	SEP 3 2013
A Regulatory Signaling Loop Comprising the PGAM5 phosphatase and CK2 Controls Receptor-Mediated Mitophagy	Chen, Guo, et al.	208	26	MOLECULAR CELL	MAY 8 2014
Mitochondrial dysfunction in the pathophysiology of renal diseases	Che, Ruochen, et al.	193	24.13	AMERICAN JOURNAL OF PHYSIOLOGY-RENAL PHYSIOLOGY	FEB 2014
miR-375 Inhibits Autophagy and Reduces Viability of Hepatocellular Carcinoma Cells Under Hypoxic Conditions	Chang, Ying, et al.	193	19.3	GASTROENTEROLOGY	JUL 2012
DUSP1 alleviates cardiac ischemia/reperfusion injury by suppressing the Mff-required mitochondrial fission and Bnip3-related mitophagy via the JNK pathways	Jin, Qinhua, et al.	186	46.5	REDOX BIOLOGY	APR 2018

China is a relatively late participant in mitophagy research, which explains the fewer number of cited papers published. Liu Lei et al.’s research paper “Mitochondrial outer-membrane protein FUNDC1 mediates hypoxia-induced mitophagy in mammalian cells”, published in NATURE CELL BIOLOGY, was the most cited. The article demonstrated that FUNDC1, a complete mitochondrial outer membrane protein, is a hypoxia-induced mitophagy receptor. The protein interacts with LC3 through its typical LC3 binding motif Y (18) XXL (21). The research further reveals that hypoxia induces the dephosphorylation of FDDC1 and enhances its interaction with LC3, thus achieving selective mitochondrial autophagy. These findings could provide helpful tips for mitochondrial quality control in mammalian cells.

### Comparison of Co-Occurring Keywords in the Field of Mitophagy

To determine the direction of research and keyword changes in the field of mitophagy, we used the VOSViewer software to analyze the distribution of co-occurring keywords. The keyword threshold was identified as 50 times, i.e., the keywords in the following results appeared in the title and abstract of the published paper at least 50 times.

**FIGURE 4:**
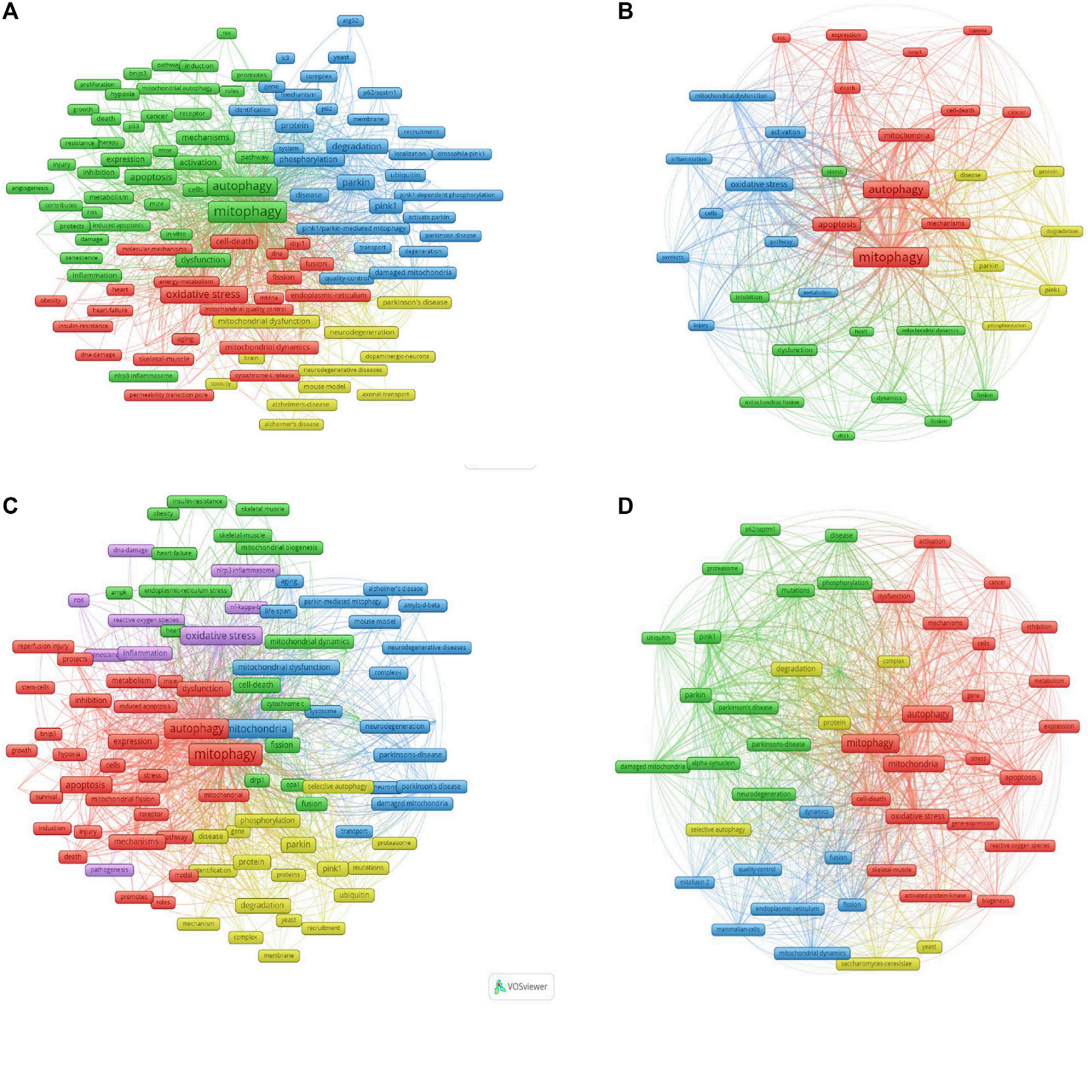
Comparison of co-occurring keywords in the field of mitophagy. **(A)** Mapping of keywords in the field of mitophagy worldwide from 2005 to 2020. **(B)** Mapping of keywords in the field of mitophagy in China from 2005 to 2020. **(C)** Mapping of keywords in the field of mitophagy from 2016 to 2020. **(D)** Mapping of keywords in the field of mitophagy from 2005 to 2015.

Figure 4 depicts 176 identified keywords that met the threshold and were classified into 4 clusters: “mitophagy”, “parkin”, “oxidative stress”, and “neurodegeneration” (Figure 4A). Figure 4B shows 37 identified keywords uncovered by the keyword co-occurrence analysis of papers in China categorized into 4 clusters: “mitophagy”, “parkin”, “oxidative stress”, and “Mitochondrial dynamics”.

Comparing the 16 years that spanned research on this field in two phases, we found that the keywords also changed dynamically over time. In reality, most of the papers on mitophagy were published in the last 5 years. In general, the burgeoning status of the research on mitophagy has seen an increase in the co-occurrence of keywords. Compared to earlier mitophagy research, investigations in the last 5 years (2016–2020) were centered on “nlrp3 inflammasome”, “skeletal-muscle”, and “heart failure” (Figure 4C). Early-stage mitophagy research (2005–2015) had relatively fewer research hotspots (Figure 4D).

## Discussion

In this bibliometric analysis, we systematically retrieved and analyzed papers published in the field of mitophagy in the past 16 years and compared the research progress and gap in this field between Chinese and foreign research institutions. This exercise provides an understanding and the ability to track development trends and research hotspots.

In the past 16 years, research on mitophagy has seen continuous and rapid development worldwide. The growth of science cannot be achieved without strong economic strength. Therefore, it does not take a genius to see that the United States has the upper hand in this field. Its institutions have published the most papers and have the highest citations and H indexes. The imbalance of global economic power has resulted in disproportionate scientific research and progress. Developed countries are leading the global trend in the research on mitophagy. However, China’s rapidly rising economic power has now seen great improvements in this field. Still, the comparison between papers published by institutions in other major countries and Chinese establishments suggests that the quality of papers published by the latter must be improved. Nevertheless, collaborations between China and the rest of the world are becoming a frequent occurrence, and many significant findings have been published in the most important journals in the field of autophagy, which contributes to the improvement in research on mitophagy in China. With growing attention and official support, progressively more investigations have been funded in this field in China, also guaranteeing China’s continuous and steady progress. From the first papers published in 2005 to 1,156 papers published in 2020, remarkable achievements have been made in the field of mitophagy worldwide, providing new perspectives for the prevention and treatment of many major diseases.

Based on these studies and results, keywords on mitophagy have also changed. For example, “Exendin-4”, a glucagon-like peptide-1 (GLP-1) receptor agonist, is a new keyword in this field. Meanwhile, it is also currently considered to be an effective treatment for type 2 diabetes mellitus (Schonhoff and Harms, 2018). Many studies have shown that this GLP-1 receptor agonist has neuroprotective effects in animal models of Parkinson’s disease (Hölscher, 2018). Exendin-4 has also shown favorable protective effects in patients with Parkinson’s disease in a phase Ⅲ clinical trial (Vijiaratnam et al., 2021). Moreover, Exendin-4 can improve motor disorders in Parkinson’s mice and protect dopaminergic neurons in the substantia nigra and striatum by reducing α-Syn accumulation and inhibiting mitophagy pathways. In animal models of nonalcoholic fatty liver disease, we found that GLP-1 receptor agonists protected the livers of mice with nonalcoholic fatty liver disease by enhancing the liver autophagy/mitophagy pathway, reducing oxidative stress damage, and inhibiting NLRP3 inflammasomes (Yu et al., 2019).

Recently, SIRT3 has received more and more mention in the field of mitophagy. SIRT3 is a class III histone deacetylase (HDAC) and a part of the sirtuin gene family predominantly located in mitochondria. It is highly expressed in the brain and could eliminate reactive oxygen species, inhibit cell apoptosis, and prevent the formation of cancer cells (Yang et al., 2019). SIRT3 reportedly has a profound effect on nuclear gene expression, cancer, cardiovascular disease, neuroprotection, aging, and metabolic control (Yu et al., 2017; Lu et al., 2019; Zhou et al., 2019). According to Yu et al., SIRT3 deficiency aggravates diabetic cardiac dysfunction. Their results also suggested that inhibiting mitophagy down-regulation mediated by SIRT3-FoxO3A-Parkin signaling could play a role in the pathogenesis of diabetic cardiomyopathy (Yu et al., 2017). We have also revealed in the past that SIRT3-mediated mitophagy reduces the apoptosis of tumor cells in hypoxia environments, which provides new ideas for tumor prevention and treatment (Zhou et al., 2019). Furthermore, for non-alcoholic fatty liver disease, SIRT3 overexpression can protect liver cells by promoting BNIP3-mediated-mitophagy and reducing mitochondrial apoptosis, thus effectively treating non-alcoholic cirrhosis (Li et al., 2018).

Along with using the keyword co-occurrence analysis, many researchers have predominantly used “nlrp3 inflammasome” for several years. NLRP3 inflammasome is an essential component of innate immunity that can be activated by multiple types of pathogens or danger signals, such as potassium ions, mtROS, and lysosomes. NLRP3 inflammasome is key to a variety of disease processes: from the first identified familial recurrent auto-inflammatory responses to type 2 diabetes, Alzheimer’s disease, and atherosclerosis (Yu et al., 2017; Li et al., 2018; Liu et al., 2019; Lu et al., 2019). Several papers have also associated the SIRT3-mitophagy-NLRP3 axis with these diseases, and it is becoming a potential therapeutic target. Per Traba et al.’s clinical investigation, fasting ameliorates the NLRP3 inflammasome activation, in part through SIRT3-mediated mitochondrial homeostatic control. The authors also suggested that deacetylase-dependent inflammasome decay could be targeted in human diseases, reflecting the prominence of the research on mitophagy currently (Traba et al., 2015).

Over the last few years, the remarkable progress in the field of mitophagy in China has correlated massively with the country’s economic strength and huge investment in scientific research. Research on mitophagy in China now involves a progressively increasing number of diseases, providing a new perspective and a fresh thinking approach in the battle for the prevention and treatment of related diseases.

Still, the gap between China and world leaders in the field cannot be ignored. While the total number of papers published by Chinese institutions has increased significantly in recent years, the frequency of citation and H-indexes remain low compared to those of its counterparts. In our bibliometric analysis, the gap between China and major countries in the world is also reflected in the journals in which the papers are published. Many papers in China are published in highly recognized journals, such as AUTOPHAGY. But these journals are mostly foreign journals. Therefore, if China wants to expand the influence of its research, it must also seek to establish recognized international journals in the field of mitophagy that can attract reputable research submissions and encourage academic cooperation.

Furthermore, we identified a substantial amount of publications on traditional Chinese medicine (TCM), which provides a unique opportunity for the development of mitophagy and traditional Chinese medicine research in tandem in China. TCM has a long history in China, and many TCM compounds are considered promising enough to be developed into approaches to treat cardiovascular and neurological diseases. However, due to the unclear molecular target, incorrect dosage, and high toxicity, the development of TCM has stagnated. Perhaps Chinese researchers combining TCM pharmacology with mitophagy could provide a remarkable opportunity to uncover promising breakthroughs in the prevention and treatment of some major diseases.

Admittedly, there are some limitations to our analysis. Firstly, bibliometric analyses cannot reflect the quality of published papers fully. And the number of citations and H-indexes only reflect the quality of a paper from a certain aspect. Secondly, the WOS database is known for its considerably slow action in adding already existing data to its database; hence, we may not have scrutinized all already published papers not yet included in the WOS database. Thirdly, only English papers were assessed, and no retrieval analysis was conducted for papers published in other languages. Additionally, we only used the VOSViewer software for keyword co-occurrence analysis, even though many bibliometric analysis tools exist. Interpretations of our findings must, therefore, be carried out with caution.

## Conclusion

The US and its research institutions have published the most papers, while the most common research category is cell biology. And AUTOPHAGY is the most popular journal for publishing most studies on mitophagy. We have also listed the most cited literature worldwide and in China, with the latter emerging as an increasingly powerful force in research on mitophagy, as state funding has increased, albeit still with a gap to close to major countries. This analysis presents a comprehensive representation of the state of research on mitophagy reflecting the development and trend in the field, which should help guide researchers in selecting topics.
